# Risk factors for delayed extubation after pediatric perineal anaplasty in patients less than 1 year of age: a retrospective study

**DOI:** 10.1186/s12887-024-04781-4

**Published:** 2024-05-06

**Authors:** Qianqian Zhang, Jing Xu, Qinghua Huang, Tianqing Gong, Jia Li, Yu Cui

**Affiliations:** Department of Anesthesiology, The Affiliated Hospital, School of Medicine, UESTC Chengdu Women’s & Children’s Central Hospital, Chengdu, 610091 China

**Keywords:** Risk factors, Delayed extubation, Perineal angioplasty, Anesthesia

## Abstract

**Background:**

Anorectal malformation is a common congenital problem occurring in 1 in 5,000 births and has a spectrum of anatomical presentations, requiring individualized surgical treatments for normal growth. Delayed extubation or reintubation may result in a longer intensive care unit (ICU) stay and hospital stay, increased mortality, prolonged duration of mechanical ventilation, increased tracheostomy rate, and higher hospital costs. Extensive studies have focused on the role of risk factors in early extubation during major infant surgery such as Cardiac surgery, neurosurgery, and liver surgery. However, no study has mentioned the influencing factors of delayed extubation in neonates and infants undergoing angioplasty surgery.

**Materials and Methods:**

We performed a retrospective study of neonates and infants who underwent anorectal malformation surgery between June 2018 and June 2022. The principal goal of this study was to observe the incidence of delayed extubation in pediatric anorectal malformation surgery. The secondary goals were to identify the factors associated with delayed extubation in these infants.

**Results:**

We collected data describing 123 patients who had anorectal malformations from 2019 to 2022. It shows that 74(60.2%) in the normal intubation group and 49(39.8%) in the longer extubation. In the final model, anesthesia methods were independently associated with delayed extubation (*P* < 0.05).

**Conclusion:**

We found that the anesthesia method was independently associated with early extubation in neonates and infants who accepted pediatric anorectal malformation surgery.

## Introduction

Anorectal malformation is one of the general congenital problems occurring in approximately 1 in every 5,000 births and presenting with anatomical variations that require individualized surgical treatment to ensure normal growth [1]. Low anorectal malformation/rectoperineal fistula may be ignored in the newborn. When symptomatic, it may be corrected by a simple angioplasty with excellent results [2]. Early tracheal extubation after anesthesia can help rapid intestinal recovery and restore the normal growth and development of infants, as well as improve infant outcomes and lower postoperative complications [3–5]. Among surgical interventions for infants, anesthesia-related critical events occur at a high incidence rate of up to 3.4% [6], with respiratory events being particularly common(46.5%) [7]. For neonates and small infants under anorectal malformation surgery, anesthesiologists’ decisions on the appropriate time of extubation after pediatric gastrointestinal surgery are often challenging from many aspects with regard to complex and rapidly deteriorating situations such as airway obstruction, hypoxemia, and possibly urgent reintubation. However, delayed extubation or reintubation may result in longer intensive care units (ICU) and hospital stays, increased mortality, prolonged duration of mechanical ventilation, increased tracheostomy rate, and higher hospital costs [8]. Therefore, it is necessary and beneficial to postoperative early extubation for infants.

Extensive studies have focused on the role of risk factors in early extubation during major infant surgery such as cardiac surgery, neurosurgery, and liver surgery [9–12]. Many studies demonstrated that there is a significant difference in the proportion and duration of postoperative respiratory for neonates and infants. Wang et al. summarized that for 253 newborns who underwent gastrointestinal surgery, the average length of postoperative mechanical ventilation was 3 days [13]. A clinical study conducted by Ceelie et al., included 71 newborns and infants undergoing thoracic and abdominal surgery without cardiac surgery, abdominal surgery accounted for 77.5%, of which 40.8% required mechanical ventilation after surgery with a duration of 23–34 h [14]. However, few articles have mentioned that other minor and medium surgeries also need early extubation for infants. The authors describe their experience of maintaining spontaneous breathing in neonates undergoing Fast-track anesthesia (FTA group) which does not use opioids or muscle relaxants during anoplasty. Extubation time in the FTA group was significantly shorted [15]. However, no study has mentioned the influencing factors of delayed extubation in neonates and infants in neonatal and infant angioplasty surgery.

The principal goal of this study was to investigate the incidence of delayed extubation in pediatric anorectal malformation surgery. The secondary goal was to identify the factors associated with delayed extubation in these patients. Results can help achieve early extubation by providing evidence on optimizing clinical decision-making and improving outcomes in those patients.

## Materials and methods

### Study design and participants

This is a single-center, retrospective case-control study, which has been approved by the Institutional Review Board (IRB) of Chengdu Women’s and Children’s Central Hospital [No. 2022 (100)].

The need for Informed Consent was waived by the Institutional Review Board (IRB) of Chengdu Women’s and Children’s Central Hospital due to the retrospective nature of the study. The study adheres to the applicable Strengthening the Reporting of Observational Studies in Epidemiology (STROBE) standards for observational studies. This study enrolled infants with anorectal malformation who were scheduled for angioplasty, either elective or emergency. Clinical protocol is readily available for all general procedures and can be accessed at each anesthesia station through the Department of Anesthesiology intranet.

### Patient population

Inclusion criteria are data for infants who had anorectal malformation surgery between June 2018 and June 2022. Eligibility criteria included neonates and infants (≤ one-yea*r-*old) who were diagnosed with anorectal malformations and underwent surgical intervention (i.e., angioplasty), scheduled for either elective or emergency.

Infants who were dependent on mechanical ventilation preoperatively; lacked birth or hospital records; had received major surgery involving vital organs oncomitantly, and had major cardiopulmonary diseases were excluded. The infants and newborns with contraindications for caudal anesthesia were excluded. Major cardiac defects were defined as any congenital heart anomaly that requires surgical interventional or cardiac catheterization before discharge from the neonatal intensive care unit (NICU) or pediatric intensive care unit (PICU).

All cases were performed by attending anesthetists with ≥ 5 years of experience in pediatric anesthesia. As a rule in the authors’ institution, only anesthetists with ≥ 5 years of experience attending in pediatric anesthesia can perform anesthesia in neonates.

### Anesthesia management

#### Anesthesia management

The patients who were diagnosed with anorectal malformations and underwent surgical intervention received general anesthesia (GA) or general anesthesia with caudal anesthesia (GA + CA). The protocol for the management of infants is as follows. An intravenous line was established in the ward. Thermal blankets and appropriate room temperature were prepared before the patients entered the operating room. Spontaneous breathing was maintained in 100% oxygen (4 L/min) initially via a mask. Anesthesia was induced by using fentanyl, midazolam, and cis-atracurium. If the respiratory rate decreased to < 20 breaths/min, mechanically controlled ventilation was rapidly assisted. When the appropriate depth of anesthesia was achieved, endotracheal intubation was performed under a laryngoscope. All of the caudal blocks were administered following the induction of anesthesia. The procedure was performed by the experienced attending anesthetist. If the age of patients is younger than 30 days, lidocaine (1mL/kg [0.5%]) is injected after confirmation in epidural space; otherwise, lidocaine (0.5mL/kg [0.5%]) and ropivacaine (0.5mL/kg [0.2%]) will be given. Anesthesia was maintained with sevoflurane and was discontinued 3 min before the completion of surgery. Procedures in the other group were performed using traditional general anesthesia without caudal block. To ensure safety and improve surgical efficiency, the anesthesiologist decided whether to extubate the endotracheal tube in the surgical intensive care unit (SICU) or the operating room. All patients were transferred to SICU after the operation.

### Hemodynamic monitoring

All patients were routinely monitored. In the operation room, electrocardiograph (ECG), non-invasive blood pressure, end-tidal carbon dioxide (ETCO2), pulse oximetry, and body temperature were continuously monitored.

#### Group and definition of delayed extubation

Additionally, postoperative data including the duration time from the completion of anesthesia to tracheal extubation were classified into two categories: normal(< 6 h) and long extubation (≥ 6 h) [16]. Lastly, all adverse events were recorded, such as reintubation within the subsequent 24 h.

The indicators of extubation in infants encompassed full consciousness by limb movement, regular breathing, and tidal volume 5–8mL/kg. Perioperative data were retrieved from the database [17]. 

#### Variables

Preoperative data collected included age, gender, gestational weeks, birth body weight, diagnosis, prematurity history, surgery and intubation history, coexisting cardiac anomalies, coexisting respiratory diseases, failure to thrive, developmental retardation, American Society of Anesthesiologists (ASA) classification, preoperative oxygen supplements, preoperative hemoglobin.

Intraoperative factors included anesthesia methods, duration of surgery, duration of anesthesia, furosemide exposure, estimated blood loss, fluid infusion and urinary output, blood transfusion, and time of extubation. We also collected the patient’s intraoperative opioids, muscle relaxant drugs and antagonists, and other drug consumption.

Postoperative data collected included incidence of reintubation within 24 h; major complications including caudal anesthesia complications were defined as neurologic (paresthesias or a persistent neurologic deficit), local anesthetic (LA) systemic toxicity, infection, vascular (vascular puncture or hematoma), respiratory (pneumothorax or respiratory depression), catheter malfunction, dural puncture, or other; including death, and post-operative ICU, in-hospital mortality, readmitted within 30 days, and accepted an unplanned reoperation.

### Study outcome

The principal goal of this study was to investigate the incidence of delayed extubation in pediatric anorectal malformation surgery. The delayed extubation was defined as more than 6 h (≥ 6 h), which was in line with the study of Lamoshi A, et al. The secondary goals were to identify the factors associated with delayed extubation in these patients.

### Statistical analysis

In the analysis, categorical variables were represented by number and percentage (%), while continuous variables were represented by mean and standard deviations (mean ± SD) or median and range (min–max). Univariate analysis was used for comparing two categorical variables by chi-square test or Fishers Exact test, and two continuous variables were tested by independent sample t-test or Mann-Whitney U test. The variables associated with delayed extubation (*P* ≤ 0.1) by univariate analysis were included in the multivariate logistic regression for identification of predictive risk factors. Adjusted odds ratio and 95% CI were reported. A *P*-value of < 0.05 was considered statistically significant. All statistical analyses were performed by SPSS software, version 22.0 (IBM Corp., Armonk, NY, USA).

## Results

### Baseline characteristics

**Fig. 1:**
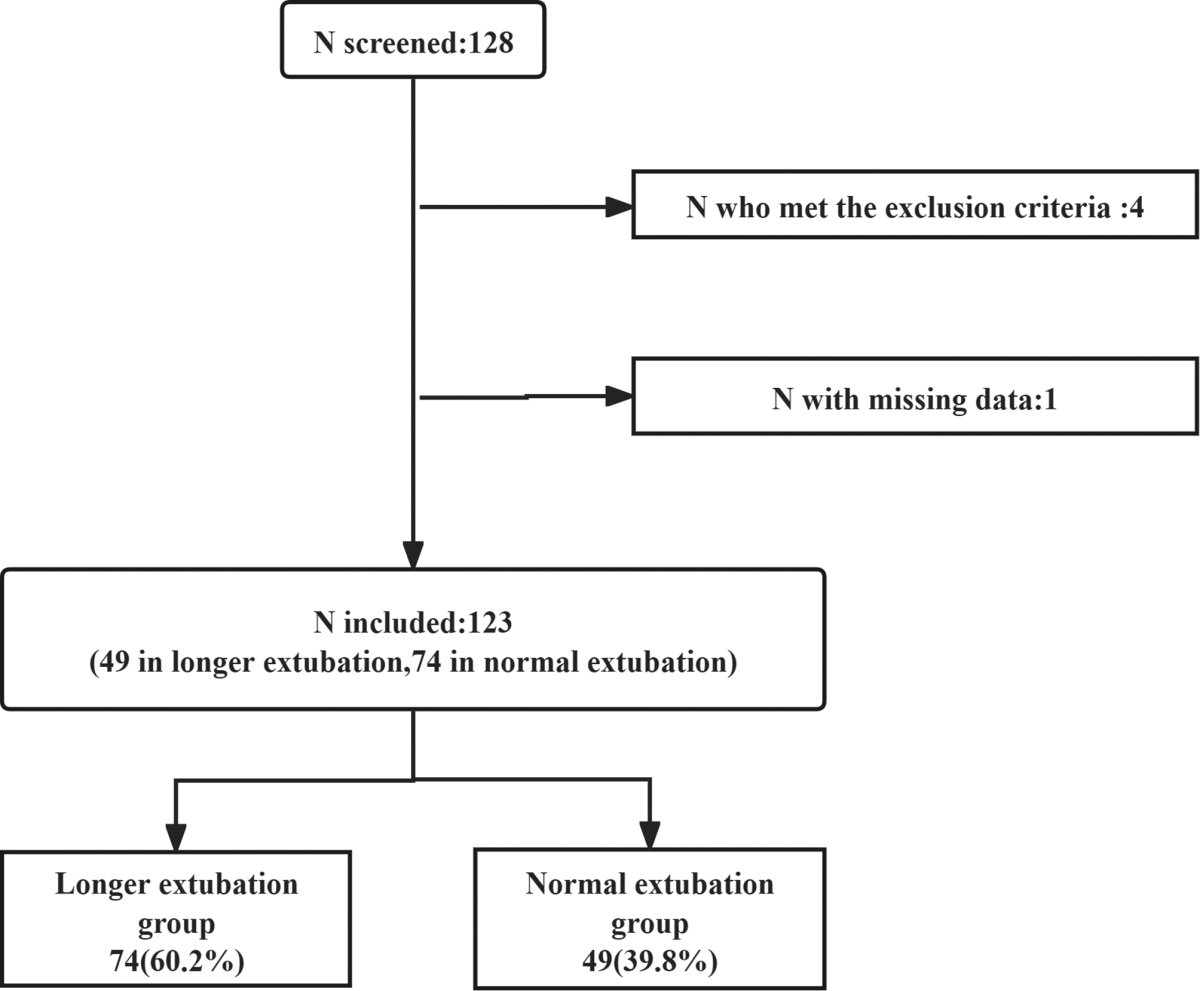
Participants flow diagram

The study flowcharts are displayed in Fig. 1. Data describing 128 patients who had anorectal malformations from 2019 to 2022 were collected. From the remaining 128 patients, 1 patient was excluded due to inadequate data; 4 patients who met the exclusion criteria were deleted. Thus, 123 patients were enrolled in the final analysis. According to the definition of delayed extubation, there were 74 (60.2%) cases in the normal intubation group and 49(39.8%) in the longer extubation group.

### Independent variables and analysis of risk factors

A total of 123 pediatric patients were included in our study. Table 1 summarizes the baseline characteristics of patients. The incidence of delayed extubation postoperative transfusion of the normal extubation group was 60.16%(74/123). Preoperative factors that had statistically significant differences were age group, body weight, intubation history, preoperative hemoglobin, and ASA (Table 1). There was no statistical significance in sex and confidence of comorbidity including PDA, anemia, and premature infants.

**Table 1:** Demographic and clinical features of patients less than one years age with pediatric perineal anoplasty

Variables	**Patients, No.(%)**		*P* value
	**Normal (n = 49)**	**LONG (n = 74)**	
Sex (Males) (n, %)	25(51)	46(62.1)	0.222
**Age**			
newborns(< 30days)	23(53.1)	69 (93.2)	< 0.01*
infants(> 30days)	26(55.8)	5(6.7)	
Weight (kg) [Median(IQR)]	4.96(2,10.5)	3.23(2.3,7.1)	< 0.01*
Preoperative HB[Median(IQR)]	139.34(71,206)	166.23(81,237)	< 0.01*
Premature	3(6.1)	6 (8.1)	0.68
Anemia	8(16.3)	5(6.8)	0.101
PDA	9(8.3)	19(25.6)	0.326
**ASA(n, %)**			
I–II	21(42.9)	9(12.1)	< 0.01*
III	28(57.1)	65(87.8)	
**Anesthesia**			
GA	22(44.8)	56(75.6)	< 0.01*
GA + CA	27(55.1)	18(24.3)	
Intubation history	7(14.2)	1(1.3)	0.021*
**Intraoperative Index**			
Anesthesia time[Median(IQR)]	140(55,310)	127(45,344)	0.224
Operation time[Median(IQR)]	89(10,215)	71(12,252)	0.066*
Tube time[Median(IQR)]	79.3(2,340)	1727(221,11482)	0.005*
Fluid[Median(IQR)]	89(20,300)	91(30,1000)	0.914
Urine output[Median(IQR)]	23(0,160)	23(0,160)	0.11
Blood loss[Median(IQR)]	3.1(0,15)	3.5(0,20)	0.51
Opioid(n, %)	47(96.0)	71(96.0)	0.994
Muscle relaxant(n, %)	38(77.5)	70(94.5)	0.009*
Muscle relaxant antagonism(n, %)	1(2.0)	0(0)	0.25
**Ventilation mode**			
SPONT(n, %)	7(14.2)	0(0)	0.999
MV(n, %)	42(85.7)	74(100)	
Postoperative complications(n, %)	2(4.0)	1(1.3)	0.36
Postoperative transfusion(n, %)	5(10.2)	10(13.5)	0.584

Note: n Sample, SD Standard deviation, IQR Interquartile Range, delayed extubation, *P < 0.1

The intraoperative factors including anesthesia methods, tube time, duration of surgery, use of muscle relaxants were statistically associated with the delayed extubation group. Duration of anesthesia, volume of blood loss, fluid, urine output, and the use of opioid and muscle relaxant antagonism, ventilation mode, and comorbidity have not been statistically associated with delayed extubation (Table 2). Outcomes including postoperative transfusion and postoperative complication have no statistically significant differences (Table 2). The variables with *P* < 0.1 were entered into stepwise multiple logistic regression models to delayed extubation.

**Table 2:** Univariate and multivariate logistic analysis of risk factors related to delayed extubation in pediatric gastrointestinal surgery

Independent variables	Univariate analysis		Adjusted multivariate analysis		
	OR (95% CI)	*P* value	Adjusted OR (95% CI)	*P* value	
Characteristics					
Sex (Males) (n, %)	1.577 (0.759,3.276)	0.222			
Newborns(< 30days)	0.064 (0.022, 0.186)	< 0.01*	1.031(0.982,1.083)	0.219	
Infants(> 30days)					
Weight (kg) [Median(IQR)]	2.208(1.509,3.231)	< 0.01*	1.019(0.995,1.044)	0.342	
Preoperative HB[Median(IQR)]	0.976(0.965,0.988)	< 0.01*	1.014(0.989,1.040)	0.121	
Premature	1.353 (0.198, 2.573)	0.68			
Anemia	2.693(0.826,8.783)	0.101			
PDA	0.639(0.262,1.561)	0.326			
ASA	0.185(0.075,0.453)	< 0.01*	1.006(0.117,8.631)	0.996	
Anesthesia method	3.818 (0.1761, 8.278)	< 0.01*	3.579(1.310,9.773)	0.013*	
Intubation history	12.167(1.447,102.319)	0.021*	12.018(0.701, 205.929)	0.086	
Second surgery	2.689(0.322,5.685)	0.19			
Intraoperative Index					
Anesthesia time[Median(IQR)]	1.004(0.998,1.010)	0.224			
Operation time[Median(IQR)]	1.006(1.000,1.013)	0.066*	1.003(0.993,1.012)	0.6	
Tube time[Median(IQR)]	0.975(0.957,0.992)	0.005			
Fluid[Median(IQR)]	1.000(0.996,1.004)	0.914			
Urine output[Median(IQR)]	1.014(0.997,1.031)	0.11			
Blood loss[Median(IQR)]	0.964(0.864,1.075)	0.51			
Opioid(n, %)	1.000(0.893,1.118)	0.9944			
Muscle relaxant(n, %)	0.197(0.059, 0.662)	0.009*	0.286(0.069,1.177)	0.083	
Muscle relaxant antagonism(n, %)	0.662	0.25			
Ventilation mode	0.693	0.999			
Postoperative complications(n, %)	3.106 (0.274, 35.224)	0.36			
Postoperative transfusion(n, %)	0.727(0.233,2.274)	0.584			

Note: Clinically significant variables and those with *p*-value on univariate regression of < 0.10 were subsequently included in the multivariate regression model; 95% Confidence Intervals (95% CI), *P < 0.05 is statistically significant

In the univariate analysis, age, weight, preoperative HB, ASA status, anesthesia methods, intubation history, operation time, use of muscle relaxant, and PLOS were independently associated with delayed extubation statistically (Table 2).

The variables with *P* < 0.05 were entered into stepwise multiple logistic regression models to predict delayed extubation. Multivariable logistic regression analysis (Table 2) identified that only anesthesia methods (OR, 0.316; 95%CI, 0.105,0.9903; *P* < 0.05) were the independent risk factors for delayed extubation. These factors including Age, weight, preoperative HB, ASA status, intubation history, operation time, and use of muscle relaxant have no statistical significance for delayed extubation by stepwise multiple logistic regression models.

## Discussion

It is very important to achieve early extubation in neonates and infants who underwent pediatric perineal anoplasty [5]. Our study found that anesthesia methods were independently associated with delayed extubation.

### The incidence of delayed extubation after pediatric perineal angioplasty

Compared with adults or older children, neonates and young infants are at a higher risk for delayed extubation. The incidence of delayed extubation in preterm or high-risk infants is 17.2–35.7% in different reports; this variation could depend on the definition of delay, patient characteristics, anesthetic agents used, and whether muscle relaxant was used or not [18–20]. Obviously, the incidence of delayed extubation varies greatly in different types of surgery. Previous studies have primarily focused on delayed extubation in major pediatric surgery, such as cardiac surgery, neurosurgery, and liver surgery. For pediatric cardiac surgery, the extension of invasive mechanical ventilation time after surgery ranges from 3 to 7 days; for pediatric liver transplantation surgery, the time ranges from over 24 h to 4 days [21, 22]. Nafiu et al. proposed to define the extension of invasive mechanical ventilation time as more than 4 days, so 75% of patients are defined as delayed extubation [20]. Unfortunately, there were few studies about the incidence of early extubation after gastrointestinal surgery in neonates and infants. Our study summarized a total of 123 newborns and infants who underwent congenital anorectal surgery. Surprisingly only 49(39.8%) neonates or infants can achieve normal extubation successfully. Theoretically, congenital anorectal surgery is not a large surgery patients have a higher incidence of early extubation. Yet the incidence of delayed extubation after pediatric perineal angioplasty is 60.7% in our institution. Although theoretically, extubation is not difficult in this perineal angioplasty, in practice especially early extubation in neonates and infants less than one year is not so easy especially in non-children’s specialized hospital, despite infants undergoing uniform anesthesia management in the same institution. So the extubation of infants and newborns should be cautious during anesthesia management of non-major surgery.

### Risk factors associated with delayed extubation after pediatric perineal angioplasty

It is well known that demographic characteristics, surgical services, and intraoperative features are associated with delayed extubation. In our study, we observed a significant impact of different anesthetic techniques on the incidence of early extubation in patients with congenital anorectal. Previous research has demonstrated that general anesthesia is linked to higher perioperative adverse events such as apnea and bradycardia compared with spinal anesthesia in preterm infants receiving inguinal hernia repairs [19, 23]. Guess general anesthesia combined with caudal anesthesia (GA + CA) can reduce the requirement of anesthetics to achieve early extubation. Regional anesthesia is preferred rather than general anesthesia for preterm infants receiving the hernia surgery in early infancy with lower postoperative respiratory complications, but these comparisons mainly focused on the general anesthesia with endotracheal intubation [24–26]. Neuraxial anesthesia in neonates and infants is safe and effective [27]. One study found only 1 serious complication(meningitis) in a neonate in a series of 2490 cases [28]. Other research shown that 1 neonate of 18,650 children suffered from seizure [29]. So there were no permanent sequelae and mortality. Effective use of caudal anesthesia can provide perfect analgesic when performed properly. If patients can benefit from caudal anesthesia or region block, anesthesiologists consider applying combined anesthesia in the non-major operation of infants or newborns.

By comparison, some studies have suggested that anesthetic drugs and dosage levels might influence the determination of delayed extubation [30]. Yet results of other studies found that anesthetic techniques (inhalation or total intravenous anesthesia), nitrous oxide uses, opioid uses were not associated with delayed extubation [31]. Muscle relaxant did show statistical significance in the univariate model, but it did not retain significance in the multivariate analysis. Our study suggested that anaesthetics including fentanyl drugs had little effect on extraction time. However other studies prompt the opposite result [9]. These might be due to the heterogeneity of surgical procedures and the diversity of patients’ ages in the previous studies which are different from our population in this retrospective study.

The newborns did show statistical significance in the univariate model, but it did not retain significance in the multivariate analysis. Despite a lack of worldwide consensus, most studies have found that low birth weight (LBW) at the time of the operation and young age (< 6 months) are related to longer extubation [32]. It indicates that young age is an independent risk factor for delayed extubation after pediatric cardiac surgery [21]. The reasons are poor fatigue resistance, the endurance of respiratory muscles and immature respiratory center with poor response to hypoxia and hypercarbia [33]. However, most recent studies, including ours, have failed to find risk factors significant for delayed extubation example for age [17]. The authors found that other risk factors, in association with young age, increased the risk of delayed extubation rather than age alone. Moreover, the authors comment that it is the decision of the anesthetist and intensivist rather than the patient’s age, which is responsible for the delay in the extubation of newborns especially [33]. 

However, our finding did not demonstrate any significant association between intubation history and factors contributing to delayed extubation in pediatric patients. Nevertheless, the study revealed that children with a history of extubation experienced an increased risk of prolonged extubation time [9]. 

The potential explanation for these results is that the different institutions or countries have varying factors influencing extubation. Thus we choose a standardized approach to investigate the risk factors associated with delayed extubation in order to minimize confounding variables. These factors may guide anesthesiologists in optimizing anesthesia management for early extubation in similar surgical procedures. For example, applying region block during surgery can achieve early extubation and ensure patient safety. Scheduling extubation within office hours might enhance patient safety and effective resource utilization. Achieving early extubation in infants and newborns, not only helps in the early mobilization of the patient and reduces parental anxiety, but also lowers the cost of patient care and maximizes the utilization of limited hospital resources [33]. 

### Limitation

This study has several limitations that may limit its generalizability. Firstly, due to differences in patient characteristics between the two groups in this retrospective survey, we can only conclude that anesthesia methods may impact early extubation in infants and further randomized study is needed. Secondly in the study hospital’s independent clinical management processes and individualized decisions made by each anesthesiologist might introduce selection bias for the clinical outcomes. Thirdly the small sample size could potentially limit the validity of the conclusions, especially difficulty conduct an effective stratified analysis of the age of grouping infants and neonates. Fourthly, however, our previous work included both elective and emergency surgical intervention, which could be susceptible to selection bias. Finally, there are limited variables related to baseline clinical condition and intraoperative course included in our research, posing a high risk of unexplored confounding factors.

## Conclusion

Our study is the first analysis of risk factors associated with delayed extubation in patients less than 1 Year of Age: undergoing low anorectal malformation/rectoperineal fistula disorders. We found that type of anesthesia was independently associated with early extubation in neonates and infants who accepted pediatric anorectal malformation surgery. We wish this study can help to achieve early extubation and improve clinical decision-making.

## Data Availability

Datasets generated during and/or analyzed in the current study are available upon reasonable request to the corresponding author.

## Abbreviations

NICU: Neonatal intensive care unit
SICU: Surgical intensive care unit
PICU: Pediatric intensive care unit
FTA group: Fast-track anesthesia
HB: Hemoglobin
